# SARS-CoV-2 nucleic acid shedding is variable for every person

**DOI:** 10.1590/0037-8682-0162-2021

**Published:** 2021-07-02

**Authors:** Daiana de Lima-Morales, Priscila Lamb Wink, Fabiana Caroline Zempulski Volpato, Joíza Lins Camargo, Fernanda de-Paris, Afonso Luís Barth

**Affiliations:** 1 Hospital de Clínicas de Porto Alegre, Centro de Pesquisa Experimental, Laboratório de Pesquisa em Resistência Bacteriana (Labresis), Porto Alegre, RS, Brasil.; 2 Hospital de Clínicas de Porto Alegre, Laboratório de Diagnóstico de SARS-CoV-2 (LabCovid), Porto Alegre, RS, Brasil.; 3 Universidade Federal do Rio Grande do Sul, Faculdade de Farmácia, Programa de Pós-Graduação em Ciências Farmacêuticas, Porto Alegre, RS, Brasil.; 4 Universidade Federal do Rio Grande do Sul, Faculdade de Medicina, Programa de Pós-Graduação em Ciências Médicas, Porto Alegre, RS, Brasil.; 5 Universidade Federal do Rio Grande do Sul, Faculdade de Medicina, Programa de Pós-Graduação em Ciências Médicas: Endocrinologia, Porto Alegre, RS, Brasil.; 6 Hospital de Clínicas de Porto Alegre, Centro de Pesquisa Experimental, Porto Alegre, RS, Brasil.

**Keywords:** SARS-CoV-2, Viral RNA Shedding, RT-qPCR

## Abstract

**INTRODUCTION:**

Considering the persistent positivity on RT-qPCR tests, the results of SARS-CoV-2 were monitored to evaluate the viral RNA shedding period.

**METHODS:**

Between March and June 2020, the sequential results of 29 healthcare workers’ were monitored using RT-qPCR.

**RESULTS:**

More than 50% of the individuals remained RT-qPCR positive after 14 days. Furthermore, this is the first study to describe positive RT-qPCR for SARS-CoV-2 in a healthcare worker with mild symptoms 95 days after the first positive test.

**CONCLUSIONS:**

Sequential RT-qPCR results were heterogeneous, and the viral RNA shedding period is unique for each person.

It is well known that the transmission of severe acute respiratory syndrome coronavirus 2 (SARS-CoV-2) is marked by the spread of the virus in hospitals and mainly in the community through contact from person to person. In general, 14 days of quarantine is recommended for patients with coronavirus disease 2019 (COVID-19)^1^. Tang et al.^2^ observed that after 14 days of quarantine, approximately 10.5% of patients still presented a positive real-time reverse transcriptase polymerase chain reaction (RT-qPCR) test and hence suggested that quarantine should be extended for an additional 14 days. In our institution (Hospital de Clínicas de Porto Alegre - HCPA), it was possible to observe positive RT-qPCR results even after the 28 days of quarantine suggested by Tang et al.^2^ For this reason, the results of SARS-CoV-2 RT-qPCR of healthcare workers were monitored to evaluate the viral RNA shedding period in different people. Between March and June 2020, the sequential results of RT-qPCR for SARS-CoV-2 were evaluated in 29 healthcare workers from the HCPA with symptoms related to COVID-19 before the first test using the Center for Disease Control and Prevention (CDC) protocol^3^ ^,^ ^4^. This study used a convenience sampling of healthcare workers with two or more RT-qPCR results.

Sixteen healthcare workers (55.2%) were found to have a positive RT-qPCR result 14 days after the first test (Figure 1). After 40 days, eight healthcare workers (27.6%) remained positive for SARS-CoV-2, of which three (10.3%) were negative only after 60 days. Zhou et al.^5^ found that the nucleic acids of SARS-CoV-2 can be shed for up to 37 days in severely ill patients. However, in our study, all patients presented with only mild symptoms. 

**FIGURE 1:**
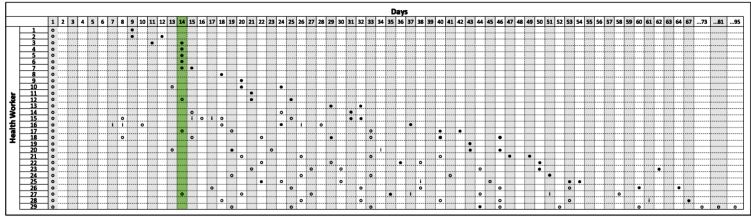
Sequential results of SARS-CoV-2 nucleic acid by RT-qPCR of the 29 healthcare workers. (○) Positive result (●) Negative result (I) Inconclusive result.

Notably, seven healthcare workers (24.1%) who presented a negative result of RT-qPCR for SARS-CoV-2 returned to present a positive RT-qPCR test after a few days. This fluctuation has also been described by other authors^6^ ^,^ ^7^. Interestingly, one patient presented a positive RT-qPCR result for SARS-CoV-2 for more than 95 days after the first test; this patient was subjected to RT-qPCR for SARS-CoV-2 11 times, one of which was negative (on day 44), but following that, six positive results were obtained. 

Initially, the CDC^1^ and the European Centre for Disease Prevention and Control (ECDC)^8^ established that healthcare workers who tested positive for SARS-CoV-2 had to remain in social isolation for 14 days and were allowed to return to work only after they tested negative by two consecutive tests of RT-qPCR for SARS-CoV-2. However, the guidelines changed after July 17, 2020, and the need for a negative RT-qPCR for SARS-CoV-2 tests was no longer required, i.e., healthcare workers who tested positive for the virus were considered able to return to work after 14 days of quarantine if they did not present symptoms for at least 72 hours.

In this study, 29 SARS-CoV-2-positive healthcare workers who presented symptoms for COVID-19 but did not require hospitalization were monitored. These individuals were evaluated after the predicted 14-day quarantine period, as well as the period they remained positive until they presented at least one, in most cases, usually two, negative RT-qPCR results for SARS-CoV-2. The exception was one healthcare worker who remained positive for 95 days and did not present a negative result, including the period of preparation of this manuscript. Moreover, in most cases (55.2%), the RT-qPCR remained positive after 14 days, and for a few patients (27.5%), the result of the molecular test remained positive beyond the 40^th^ day. According to Zhou et al.^5^, the nucleic acids of SARS-CoV-2 can be shed for up to 37 days, but this was a condition associated with severely ill patients. It should be considered that all patients included in our study presented only mild symptoms before the first exam and did not require hospitalization.

The data of our study indicate that there is a high heterogeneity in the results of sequential RT-qPCR tests of healthcare workers with mild symptoms of COVID-19 in the first test. As these individuals did not present symptoms after 14 days, it seemed unnecessary to perform the molecular test before allowing them to return to work. Our data corroborates with the last guidelines of the CDC^1^ (July 2020), which established that clinical symptoms are the most appropriate parameters to be evaluated to ensure that healthcare workers are not contagious. We see as limitation of our study the fact that the isolates were not submitted to a genetic sequencing technique. Therefore, it is not possible to guarantee that the health workers were infected by the same lineage of SARS-CoV-2 in different periods. It should also be considered that the results of the RT-qPCR cannot be directly interpreted as viral load, since the infectivity of SARS-CoV-2 (defined as growth in cell culture) can be significantly reduced when the RT-qPCR Cycle Threshold is above 24^9^. In fact, Cevik et al.^10^suggested that although patients with SARS-CoV-2 infection might have prolonged RNA shedding of up to 83 days, the live virus can be detected by culture only up to day 9 of symptoms. 

Our study is the first to describe SARS-CoV-2 nucleic acid positivity by RT-qPCR in a healthcare worker with mild symptoms 95 days after the first positive test. In fact, more than 50% of the individuals had positive RT-qPCR tests for SARS-CoV-2 after 14 days of quarantine, which indicates that the viral RNA shedding period is unique for each person. 
